# Digital literacy, ecological values, and green food consumption: an extended Theory of Planned Behavior model in Chinese universities

**DOI:** 10.3389/fpubh.2025.1723436

**Published:** 2025-12-10

**Authors:** Jiaxin He, Ke Liu, Zhenhui Ma, Zhiyu He

**Affiliations:** 1 School of International Business School, Shaanxi Normal University, Xi'an, Shaanxi, China; 2 School of Journalism and Communication, Xi'an International Studies University, Xi'an, Shaanxi, China; 3 School of Physical Education, Xi'an Jiaotong University, Xi'an, Shaanxi, China

**Keywords:** green consumption, digital literacy, ecological values, extended TPB, public health nutrition, health promotion, university students

## Abstract

**Introduction:**

Given the importance of green food consumption for public health and sustainability, understanding its drivers among university students is crucial for effective health promotion. This study investigates how digital literacy and ecological values shape green food consumption within an extended Theory of Planned Behavior (TPB) framework.

**Methods:**

We collected survey data from 836 Chinese university students. An expanded TPB model, which included digital literacy and ecological values, was developed and tested using structural equation modeling to examine their effects on consumption intentions and behaviors.

**Results:**

The model yielded three main findings. First, digital literacy was directly associated with purchase intention (*β* = 0.257, *p* < 0.001) and indirectly enhanced it by improving attitudes (*β* = 0.317, *p* < 0.001) and perceived behavioral control (*β* = 0.292, *p* < 0.001). Second, ecological values had a comprehensive impact, directly and indirectly (via attitudes, norms, and perceived control) shaping purchase intention (*β* = 0.234, *p* < 0.001). Third, digital literacy’s effect on subjective norms was non-significant (*β* = 0.069, *p* = 0.145), challenging a core tenet of the traditional TPB.

**Discussion:**

Our findings confirm that integrating digital literacy and ecological values into the TPB provides a more robust model for explaining green food consumption. Digital literacy serves as essential cognitive capital, while ecological values are foundational. The decoupling of digital literacy from subjective norms points to evolving social influence mechanisms in digital environments. This research offers practical insights for interventions and a strong theoretical foundation for future studies on sustainable dietary behaviors.

## 1 Introduction

Against the backdrop of accelerated transformation of global climate governance and the deepening of China’s “dual carbon” strategy, green consumption has evolved from an individual behavioral choice to a key mechanism for reshaping the sustainability of the economic system (1–3). The promotion of green diets, characterized by the consumption of organic, low-carbon, and sustainably sourced food (4), is increasingly recognized not only as an environmental imperative but also as a critical component of public health strategy (5–7). Unhealthy diets are a leading risk factor for the global burden of disease (8, 9), while sustainable food systems are essential for ensuring long-term food security and preventing diet-related non-communicable diseases (10).

This intersection of health and sustainability is particularly salient among China’s younger generation (11). As the backbone of the world’s largest digital consumer market (12, 13), their transition to sustainable food choices is a public health opportunity. Over 90% of this demographic participates in the digital economy through online shopping (14), and their food choices are increasingly shaped by digital environments (15). However, the digital food landscape is a double-edged sword for public health nutrition (16, 17). While it offers access to health and sustainability information, it is also rife with misinformation, algorithmic promotion of unhealthy options (18), and “greenwashing” marketing (19). Concurrently, a promising trend is emerging: young adults, particularly university students, show a greater willingness to pay a “green premium” for healthful and sustainable foods (20), a behavior linked to motivations for personal health and ecological well-being. Understanding the drivers behind these choices is thus critical for designing effective digital-age interventions to steer young consumers toward healthier and more sustainable dietary patterns, thereby addressing both public health and planetary health goals.

In public health behavior research, the Theory of Planned Behavior (TPB) has been widely and successfully applied to predict a range of health-related behaviors (21, 22), including healthy eating and vaccination uptake (23). Its core constructs—attitude, subjective norms, and perceived behavioral control, provide a robust and parsimonious framework for understanding the psychological antecedents of conscious, goal-directed behaviors (24, 25). While technology-centric models like the Unified Theory of Acceptance and Use of Technology (UTAUT) offer valuable insights into the adoption of a specific technology or system (26), the primary behavior under investigation in this study is green food consumption itself, not the use of a particular digital tool or platform. The UTAUT would be more appropriate if our focus were on explaining the adoption of a specific carbon footprint calculator app. In contrast, the TPB allows us to model how digital literacy (27), as a generalized competency, shapes the fundamental psychological building blocks (attitudes, perceived control) that lead to the health and sustainability behavior of interest. Furthermore, the TPB is explicitly designed to be open to modification by incorporating additional explanatory variables that can account for significant variance in the specific behavioral domain (28). This theoretical flexibility is crucial for our study, as it allows us to formally integrate and test the influence of two critical, era-defining factors: digital literacy, a key determinant of health literacy in navigating complex digital food environments (29). And ecological values, a fundamental motivator that aligns personal health choices with planetary health outcomes (30).

The evolution of public health research underscores the timeliness of this approach. The field is moving from a focus on individual psychology to recognizing the role of digital socio-technical systems and now to leveraging digital tools like carbon footprint apps for health and sustainability promotion (31–33). This shift highlights the “technology-value” interaction as a new frontier for public health intervention (34). Yet, critical gaps remain. It is unclear how digital literacy empowers individuals to overcome barriers (perceived behavioral control) to healthy-green food choices, or how ecological values reinforce positive health attitudes in the face of competing social pressures. Our Digital Ecological Behavior Framework (DEBF) framework addresses these gaps by formally modeling these relationships.

University students represent a strategically important population for this public health inquiry. First, as digital natives, their intensive daily use of mobile internet provides a natural setting to study how digital environments influence health decisions (35, 36). Second, they are in a critical developmental window for establishing lifelong dietary habits (37), making them a high-priority group for health promotion efforts. Furthermore, the university setting acts as a unique ecosystem where institutional food policies (38), peer influences (subjective norms), and educational exposures can be leveraged to foster sustainable healthy eating habits (39).

Therefore, this study aims to investigate the psychological mechanisms driving green food consumption among Chinese university students by proposing and validating an extended TPB model. The specific objectives are:

1. To examine the direct associates of the core TPB constructs (attitude, subjective norms, perceived behavioral control) on green food purchase intention.
2. To assess the direct influence of digital literacy and ecological values on purchase intention.
3. To investigate whether digital literacy and ecological values indirectly influence intention through the mediators of attitude, subjective norms, and perceived behavioral control.
4. To explore the interrelationship between digital literacy and ecological values.

## 2 Theoretical basis and research hypothesis

### 2.1 Theoretical foundation and an integrative lens

This study’s theoretical model rests on a fundamental premise: that information related to sustainability and green food is sufficiently pervasive and accessible within China’s contemporary digital infosphere. This premise is justified by the top-down impetus of China’s “dual carbon” strategy (carbon peak and carbon neutrality) (40), coupled with a bottom-up surge in public interest, particularly among the younger generation, in environmental and health issues (41). Government initiatives, media advocacy, the presence of environmental key opinion leaders (KOLs) on social media, and the widespread use of “green” labels on e-commerce platforms have collectively fostered a digital ecosystem rich with sustainability discourse (42, 43). It is within this context that digitally literate individuals have ample opportunity to encounter, evaluate, and process such content, thereby enabling the proposed pathways through which digital literacy may shape ecological values and subsequent green consumption decisions.

This study is primarily grounded in the TPB, which provides a robust framework for understanding the psychological antecedents of goal-directed behaviors like green food consumption. To enhance the explanatory power of our extended model and provide deeper insights into the cognitive and value-based processes at play, we also integrate perspectives from two complementary theoretical lenses: the Nature Quotient (NQ) and the Bayesian Mindsponge Framework (BMF). NQ helps conceptualize the individual differences in affinity toward nature that may underpin ecological values (44, 45). Meanwhile, BMF offers a mechanism to explain how individuals, particularly digital natives, process and internalize information in complex digital environments, thereby influencing the formation of attitudes and norms (46). These lenses will be employed post-hoc to enrich the interpretation of our findings within the TPB-based DEBF.

### 2.2 Theory of Planned Behavior and hypotheses

TPB proposed by Ajzen, aims to reveal the psychological mechanism of individual behavioral decision-making. The wide applicability of this theory has been fully verified in the field of green consumption (47, 48). According to the meta-analysis of Shen, Xu and Liu and Ma, Yin, Hipel, Li and He, TPB can fully explain environmental behavior intention (49, 50). In the context of green food consumption, behavioral attitude reflects college students’ value judgment on purchasing organic/low-carbon food. According to the cognitive-affective system theory (51), when college students associate green food with positive attributes such as “health protection” and “moral responsibility,” they form a stable attitude tendency (52, 53).

This attitude tendency is then transformed into purchase intention. Subjective norms cover two dimensions: injunctive norms and descriptive norms. For college students, injunctive norms are manifested as the clear expectations of mentors and parents for green consumption, while descriptive norms come from external demonstrations of peers’ purchasing behavior. Studies have shown that, as Du and Pan pointed out, green behavior in dormitories can not only guide environmental protection behavior and achieve energy conservation, but also have a long-term impact on sustainable development when students begin to play a role in society (54).

A critical delineation is necessary regarding the dependent variable. This study focuses specifically on green food purchase intention as the proximal antecedent to behavior. For the young, often single, university student population that constitutes our sample, purchasing decisions are overwhelmingly aligned with their own consumption (55). More fundamentally, from a public health and environmental perspective, stimulating the purchase of green food is a critical leverage point for shifting market demand and promoting sustainable food systems (56). The act of purchase, regardless of the immediate consumer, directly supports environmentally friendly production and signals market trends. Therefore, measuring purchase intention is a valid, behaviorally relevant, and strategically important dependent variable for this research.

Perceived behavioral control not only involves traditional barriers such as economic costs and product availability, such as the price premium of green food, but also includes new dimensions such as information processing ability (identifying the authenticity of green certification) and technology application ability (using price comparison tools to screen high-cost products) in digital consumption scenarios. When individuals perceive that they have the ability to overcome obstacles, they are more inclined to transform their attitudes into actual actions. Consumers’ food literacy has a strong positive impact on their willingness to consume green food (57, 58).

Al-Swidi integrated TPB, values-beliefs-norms (VBN) and cognitive-affective-behavior (CAB) theories and found that green attitudes, green social influence and green perceived behavioral control directly promoted green purchasing behavior (59). Ansu-Mensah (60) evaluated the impact of college students’ perceptions of green products on their willingness to purchase, revealing that price, value and quality are key influencing factors. Wang et al.’s study showed that goal framing theory showed a positive correlation between subjective norms, attitudes and personal norms in predicting consumers’ intention to visit green hotels (61). Saut and Saing (62) combined TPB with environmental concern and willingness to pay to analyze the willingness of Cambodian Generation Z college students to purchase environmentally friendly products, and found that attitudes toward environmentally friendly products positively associated purchase intention, and environmental concern had a significant impact on attitudes and purchase intention.

As for the penetration of subjective norms on behavioral attitudes, traditional TPB regards attitudes and subjective norms as parallel variables, but the latest research shows that social norms can reshape individual value judgments through the process of norm internalization (63, 64). In the university environment, the institutionalization of sustainable consumption education and daily interactions with peer groups will prompt students to transform external norms into internal moral standards. College students who have undergone green food training and food literacy training will have a significant improvement in their environmental protection attitudes (65, 66).

Subjective Norms capture the perceived social pressure from important referents (e.g., peers, family) regarding green food purchases (67, 68). This includes both injunctive norms (the perception that others think one *should* buy green food) and descriptive norms (69). In the collectivist and community-intensive campus environment, these norms are particularly potent (70). Behavioral attitudes includes positive associations with personal health, a value deeply rooted in Chinese cultural notions of “food as medicine” and a sense of moral responsibility toward the environment. According to the cognitive-affective system theory, when students form strong positive affective and cognitive evaluations, a stable attitudinal tendency emerges (71). This “norm → attitude” transmission path is particularly significant in the context of collectivist culture.

The TPB has been widely applied to understand and predict food consumption behavior (72, 73). Some scholars have used the TPB framework to study consumers’ willingness to purchase organic food, finding that attitude is the main driving factor (74). Other studies have shown that subjective norms can effectively predict young people’s health food choices influenced by social media (75). Research in the Chinese context has further combined TPB with food safety concerns, confirming the model’s strong explanatory power for fresh food purchasing behavior (76, 77). These studies provide a solid theoretical foundation for applying TPB to the field of green food consumption in this research. However, existing literature rarely focuses on the interaction between digital literacy and ecological values, especially among the unique group of Chinese university students.

In summary, according to the TPB model, the three key psychological factors of behavioral attitude, subjective norms and perceived behavioral control will have a significant positive impact on college students’ willingness to consume green food. Based on this, this paper proposes the following hypothesis:

*H1:* Behavioral attitude has a positive impact on college students’ willingness to purchase green food.

*H2:* Subjective norms have a positive impact on college students’ willingness to purchase green food.

*H3:* Perceived behavioral control has a positive impact on college students’ willingness to purchase green food.

*H13:* Subjective norms have a positive impact on college students’ attitudes toward purchasing green food.

### 2.3 Concept and hypothesis of digital literacy

Digital literacy, the ability of individuals to effectively acquire, evaluate, create and communicate information in a digital environment, has become a key skill in the era of information overload. Consumers with high levels of digital literacy can use algorithmic countermeasures to quickly filter out useful information, reduce the burden of decision-making, and thus improve information screening efficiency and enhance behavioral intention. These individuals can identify the actual benefits of green food, rather than being influenced solely by brand propaganda. In the field of social media, digital literacy enhances social influence through a standardized visual mechanism: active social participants can not only identify obvious norms, but also have insight into implicit normative signals such as comments and likes. Digital literacy also breaks traditional restrictions through resource substitution mechanisms. For example, when physical channels are limited, high-literacy consumers can conduct cross-regional purchases or initiate community group purchases through online platforms. Hu and Meng’s research reveals how consumer digital literacy associates green consumption behavior through self-efficacy, outcome expectations, anticipated pride, and anticipated guilt (78). Zolfaghari et al.’s research shows that the improvement of consumers’ digital purchasing power indirectly enhances the attractiveness of online shopping, thereby increasing their willingness to adopt new shopping methods (79). In addition, Severo et al. found in a Brazilian sample study that frequent use of social media can positively influence environmental awareness, sustainable consumption awareness, and social responsibility awareness (80).

*H4:* Digital literacy has a positive impact on college students’ willingness to purchase green food.

*H5:* Digital literacy positively associates college students’ attitudes.

*H6:* Digital literacy positively associates college students’ subjective norms.

*H7:* Digital literacy positively associates college students’ perceived behavioral control.

### 2.4 Concept and hypothesis of ecological values

Ecological values refer to an individual’s fundamental cognitive orientation toward the relationship between humans and nature, which determines their behavioral priorities in environmental issues (81). Different from traditional one-dimensional measurements of environmental concern, this study integrates Schwartz’s value bipolar model with conclusions from Chinese localization research (82) and deconstructs it into three operational dimensions. This framework captures the unique logic of “government-market-society” collaborative governance in the Chinese context (83):

(1) Self-interest orientation, focusing on direct benefits of green consumption on personal health (e.g., disease prevention functions of organic food) (75, 84, 85);
(2) Altruistic orientation, emphasizing intergenerational responsibility and ecological justice (e.g., reducing carbon footprints to protect future generations’ survival rights), reflecting moral initiative in social cognitive theory (86);
(3) Institutional trust, reflected in reliance on government certification systems and policy incentives for sustainable consumption (e.g., recognition of green food labels) (87).

As a digital native and policy-sensitive group, college students’ ecological values show three generational characteristics. First, health-driven egoism. Influenced by fitness culture and social anxiety, college students tend to regard green food consumption as self-investment (88, 89). Carfora’s research shows that the primary motivation for students to choose organic food is to prevent sub-health (90). According to the research results of Yang et al., college students’ health values and health awareness have a significant impact on healthy eating beliefs, which in turn have a positive impact on personal norms and consequence awareness (91). Second, altruistic tendencies under digital empowerment. Social media platforms have significantly enhanced young people’s awareness of ecological citizenship (92). Chen, S. and other researchers adopted a two-stage research design and used “Ant Forest” as an example to confirm the positive role of gamification elements in digital empowerment in shaping user values, thereby promoting the implementation of environmental protection behaviors. In addition, the study also revealed that values and environmental awareness play a role in sequential regulation between gamification functions and green consumption behaviors (93); Third, the contradiction of institutional trust. Although college students recognize the authority of government green certification. However, due to the information overload on e-commerce platforms, the problem of confusion in certification marks is particularly prominent (94). Such conflicts reflect the complex mentality of coexistence of trust and suspicion (95, 96).

Obviously, green purchasing behavior plays a vital role in pro-environmental behavior at the individual level, and it is closely related to the ecological values internalized by consumers. If consumers uphold ecological values, they will systematically consider the potential negative impact of their behavior on the ecological environment in the process of making purchasing decisions. On this basis, green products that contain energy-saving and environmentally friendly characteristics are more in line with the consumption concepts and expectations of such consumers, thereby effectively stimulating their strong motivation to purchase green products. This phenomenon shows that ecological values play a decisive role in shaping consumers’ green purchasing behavior, and it is a key psychological driving factor in promoting sustainable consumption patterns. Therefore, this study proposes the following hypothesis:

*H8:* Ecological values have a positive impact on college students’ willingness to purchase green food.

*H9:* Ecological values positively influence college students’ attitudes.

*H10:* Ecological values positively influence college students’ subjective norms.

*H11:* Ecological values positively influence college students’ perceived behavioral control.

### 2.5 The relationship between digital literacy and ecological values

In the context of social digitalization and sustainability, the relationship between digital literacy and ecological values presents a spiral upward feature of technological empowerment and value internalization (97, 98). With the advancement of digital technology, individuals not only gain stronger technical capabilities, but also gradually internalize sustainable values in the process of information acquisition and processing (99), forming a mutually reinforcing interactive relationship between the two.

Based on information ecology theory, digital literacy is not only a technical ability, it also involves the ability to screen, judge and apply information. Under this framework, college students can significantly improve their ecological cognition level by screening and processing environmental information through digital technology. Individuals with high digital literacy can effectively identify authoritative sources of environmental information (100). And by comparing data from different channels, they can form systematic ecological cognition, thereby promoting the internalization of their ecological values.

In addition, the interaction and value resonance of virtual communities in digital space further strengthen this internalization process. Individuals with higher digital literacy are better at capturing and responding to environmental value signals in the group. Through interaction with other group members, these individuals can not only strengthen their green consumption awareness, but also shape their own environmental identity in community recognition. Therefore, based on the above analysis, this study proposes the following hypothesis:

*H12:* Digital literacy has a positive impact on ecological values.

**Figure 1:**
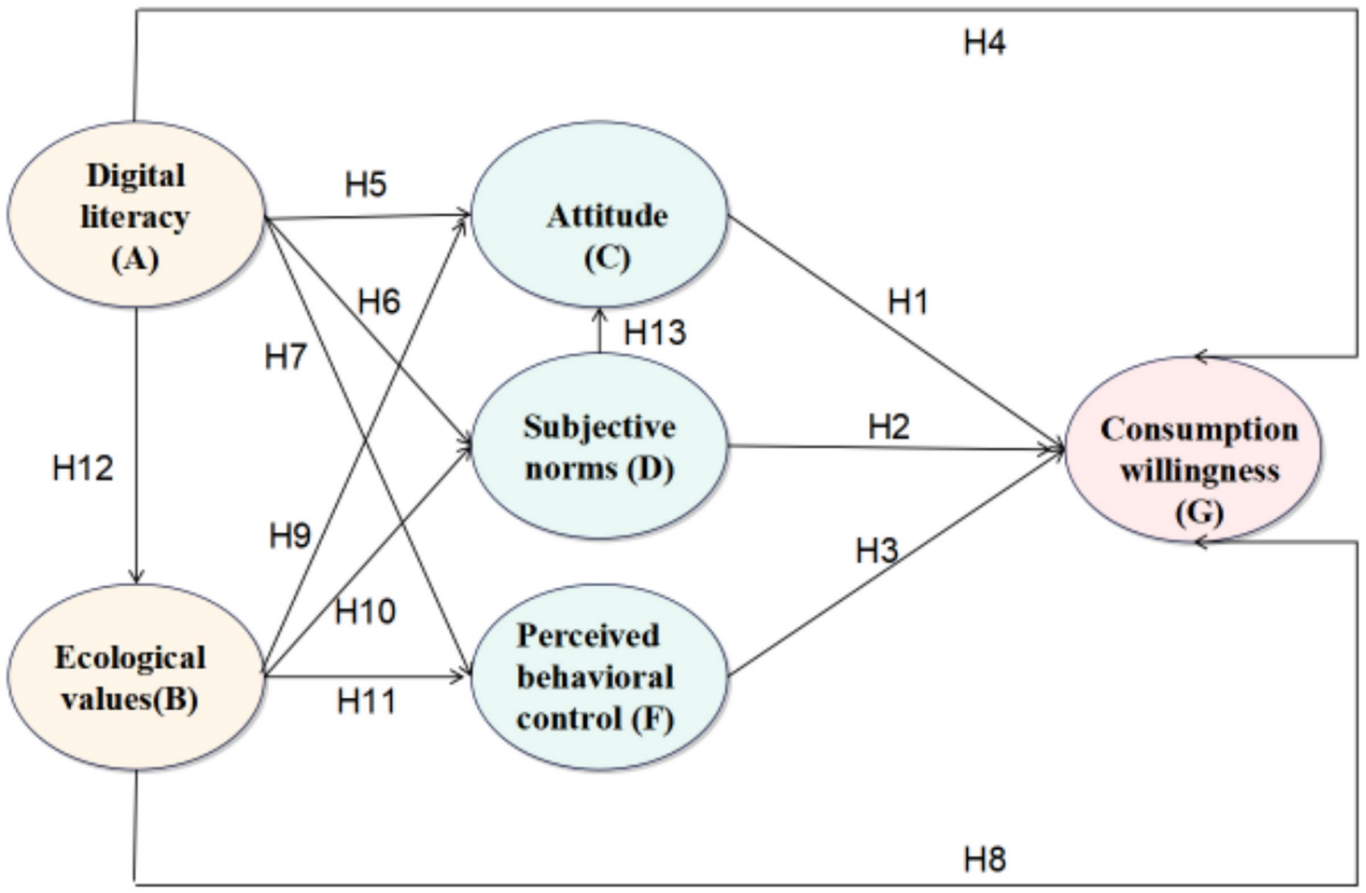
Influence model of college students’ green food consumption willingness.

To sum up, the framework diagram of the research model in this paper (Figure 1).

## 3 Study design

### 3.1 Scale and questionnaire

The theoretical model of this paper involves six variables, and all the scales use a five-level Likert scale, ranging from “strongly disagree” to “strongly agree.” The measurement questions of all variables are derived from well-established scales in existing studies, and are appropriately modified in the context of green food purchase intention to obtain Table 1.

**Table 1:** Definitions and descriptions of measurement items.

Variable	Code	Item	Source
Digital literacy (A)	A1	I can use devices such as smartphones and computers.	Reddy et al (118)
	A2	I will promptly use relevant equipment to record and collect data or information.	
	A3	I believe that obtaining information through internets can meet my daily needs of production and life.	
	A4	I’m able to interact and share information online skillfully	
Ecological values (B)	B1	I think protecting the environment is everyone’s responsibility, especially for our generation.	
	B2	I am willing to pay more for eco-friendly or green foods because I believe they have a positive impact on the environment.	
	B3	I think my daily consumption behaviors (such as buying green food) contribute to environmental protection.	
	B4	I will actively choose green foods even if they are more expensive because I value environmental protection.	
Behavioral attitude (C)	C1	Do you think buying green food is a good idea?	Van de Velde et al. (119)
	C2	Based on all your food consumption experiences, do you think buying green foods is a wise choice?	
	C3	Do you think green food consumption can contribute to environmental protection and sustainable development?	
	C4	Are you sincerely satisfied with your green food consumption experience?	
Subjective norm (D)	D1	Friends and family members around me support buying green food	Ha and Janda (120)
	D2	Friends and family around me often buy green food	
	D3	Friends and relatives around me are more satisfied after buying green food	
	D4	Your friends and family want you to buy green food	
Consequence Perception (F)	F 1	The production of unsustainable food causes serious pollution and damage to the environment	Yeung and Morris (121)
	F 2	Unsustainable food generates high energy consumption and carbon emissions, which can cause environmental pollution	
	F 3	Unsustainable food production puts the health of nearby residents at risk	
	F 4	Subsequent processing of non-sustainable food will pollute the environment.	
Purchase intention (G)	G 1	I am happy to buy green food	Rausch and Kopplin (122)
	G 2	I plan to buy more green foods in the future	
	G 3	I will continue to choose to buy green food in the future	
	G 4	I am willing to recommend green food to my relatives and friends	

### 3.2 Data and samples

This study uses online and offline consumer questionnaire data. The survey subjects are college students from various universities in China. The offline questionnaire is used for a small-scale pre-survey to ensure the rationality of the questionnaire, while the online questionnaire is used to expand the sample size and sample range. We received a total of 900 questionnaires. Eliminating samples that have not purchased green food and incomplete and after eliminating samples from respondents who reported never having purchased green food, as well as incomplete and carelessly completed responses (e.g., those with obvious straight-lining patterns). We finally obtained 836 valid questionnaires. In order to ensure that the questions are set reasonably and the respondents can understand them clearly, we conducted a small-scale pre-survey in a university in Xi’an, Shaanxi after the first draft of the questionnaire was completed. According to the feedback from the respondents, we found that the selection of each main indicator and the design of the questionnaire length were reasonable, but some professional terms were too many, resulting in the respondents’ unclear or ambiguous expressions. The 836 valid questionnaires analyzed in this study come exclusively from the subsequent, independent online survey. This separation ensures that no respondent was influenced by prior exposure to an under-developed instrument.

Therefore, we modified the item expressions that caused difficulties or ambiguity, improved the rationality and scientificity of the questionnaire, and thus formed the final questionnaire. The measurement scales for this study were adapted from well-established instruments in the literature to ensure content validity. All constructs were measured using multiple items on a five-point Likert scale. A rigorous adaptation process was undertaken to ensure the scales’ appropriateness for the context of green food purchase intention among Chinese university students. First, contextual adaptation was performed. The wording of the original items was modified to specifically reference “green food” instead of broader terms like “eco-friendly products.” This refinement precisely framed the questions within our research domain and enhanced respondent comprehension. Second, the instrument underwent pretesting and refinement. A preliminary draft of the questionnaire was administered to a small group of university students in a pilot study (43 questionnaires). Feedback revealed that some technical terms were overly complex and caused ambiguity. Consequently, we simplified the language and refined the item phrasing to improve clarity and cultural relevance for the target population, resulting in the final version of the questionnaire. The study focused primarily on the psychological constructs of interest; basic demographic information (gender) is reported, while other demographic factors were not the focus of this analysis. Finally, the final measurement items and their sources are summarized in Table 1. Following data collection, the adapted instrument was comprehensively validated, with the results reported in Section 4.1.

The survey subjects are college students in various universities in China. To ensure national representativeness and geographical diversity, this study employed a stratified sampling strategy across China’s eastern, central, and western regions. Data collection was strategically focused on the western region, including students from diverse institutions in Shaanxi province such as Shaanxi Normal University, Northwest University, Xi’an Jiaotong University, Xi’an University, and Xianyang Vocational Technical College. To capture a balanced national view, the sample was extended to key universities in the eastern region (Nanjing Tech University, Shandong Normal University) and the central region (Shanxi Agricultural University), reflecting varied socio-economic and developmental contexts. The distribution time is from June to September 2025. Given the large number of questions in the formal questionnaire, a clear instruction page was provided before the questionnaire begins to ensure response quality According to the statistical results, males account for 48.3% and females account for 51.7%.

### 3.3 Ethical considerations

We employed a non-probability convenience sampling method. The online questionnaire was developed using the Wenjuanxing platform, a widely used professional online survey tool in China. Another portion was completed online as a PDF. We distributed questionnaire links through various channels to reach university students from diverse backgrounds across different schools, including WeChat groups, online student support groups, and student sports groups.

This study was conducted in accordance with the ethical principles outlined in the Declaration of Helsinki. Prior to participation, all respondents were provided with a detailed digital informed consent form. This form clearly stated the research purpose, assured participants of the voluntary and anonymous nature of their involvement, and explained that the data would be used solely for academic purposes. Proceeding to the questionnaire served as their implied consent. No personally identifiable information was collected at any stage. To compensate participants for their time, they were informed that they would receive a small monetary incentive upon complete and valid submission of the questionnaire. The study protocol, focusing on anonymous survey data collection with minimal risk, was deemed exempt from full ethical review approval by the institutional guidelines at the authors’ university.

## 4 Empirical analysis

### 4.1 Reliability and validity analysis

Validity refers to the effectiveness of measurement, indicating the accuracy with which a measurement tool captures the targeted characteristics and whether the evaluation system aligns with the research objectives. Four types of validity are commonly assessed: construct validity, content validity, convergent validity, and discriminant validity. For construct validity, Kerlinge (101) suggested exploratory factor analysis (EFA) as the most effective testing method, which requires Bartlett’s test of sphericity (*p* < 0.01) and a KMO value > 0.7 to confirm the suitability of data for factor analysis.

#### 4.1.1 Construct validity

EFA was performed on the 24 items using principal component analysis with Varimax rotation. The results showed a KMO measure of 0.918 and a significant Bartlett’s test of sphericity (*χ*^2^ = 4,430.476, *p* < 0.001), confirming the data’s high suitability for factor analysis (Table 2). Six factors with eigenvalues >1 were extracted, collectively explaining 71.261% of the total variance (Appendix 1). The rotated component matrix (Appendix 2) indicated all items loaded significantly (> 0.6) on their intended constructs without cross-loadings, demonstrating a clear factor structure consistent with our conceptual model.

**Table 2:** KMO and Bartlett’s test.

KMO and Bartlett’s test	Types of statistics	Statistical values
KMO sampling suitability measure		0.918
Bartlett’s test of sphericity	Approximate Chi-Square	4,430.476
	Degrees of freedom	276
	Significance	0.000

We also used principal component analysis (PCA) in SPSS 27.0 software to conduct a principal component factor analysis on the 24 observed variables, extracting six principal components. These six components collectively accounted for 71.261% of the total variance, which exceeds the commonly accepted threshold of 50% for acceptable factor validity (102). The above analysis indicates that both the reliability and construct validity of this questionnaire are relatively valid (Appendix 1).

The questionnaire data were analyzed using SPSS 27.0 software. The Kaiser normalization maximum variance method was used. After six iterations, the data converged and seven components were extracted. The extracted factors were consistent with the questionnaire design items. The factor loadings were all greater than 0.6 and the distribution was reasonable. The rotated component matrix (Appendices 2, 3).

A Confirmatory Factor Analysis (CFA) was subsequently conducted using AMOS software to further validate the measurement model. The model fit indices suggested a good fit to the data: *χ*^2^/df = 3.403, GFI = 0.921, IFI = 0.954, TLI = 0.946, CFI = 0.954, and RMSEA = 0.054 (Table 3), all meeting the recommended thresholds for acceptable model fit. Following the confirmation of model fit, convergent and discriminant validity were assessed.

**Table 3:** Measurement model goodness of fit table.

Index	Test results	Reference standards
CMIN/DF	3.403	1–3 is excellent, 3–5 is good (123)
GFI	0.921	>0.9 is excellent, >0.8 is good (121)
IFI	0.954	>0.9 is excellent, >0.8 is good (124)
TLI	0.946	>0.9 is excellent, >0.8 is good (125)
CFI	0.954	>0.9 is excellent, >0.8 is good (120)
RMSEA	0.054	<0.05 is excellent, <0.08 is good (120)

Convergent validity was established through three key criteria. First, as shown in Table 4, all standardized factor loadings were statistically significant and exceeded the 0.6 threshold. Second, the Composite Reliability (CR) values for all constructs ranged from 0.818 to 0.924, surpassing the 0.7 benchmark, indicating high internal consistency. Third, all Average Variance Extracted (AVE) values were above 0.5 (ranging from 0.530 to 0.753), confirming that each construct adequately explained the variance in its respective items.

**Table 4:** Convergent validity analysis of latent facet indicators.

Variable	Index	Estimate	SE	*p*	CR	AVE	Cronbach’s alpha
A	a4	0.806			0.896	0.682	0.896
	a3	0.809	0.039	25.819			
	a2	0.837	0.039	26.945			
	a1	0.851	0.039	27.494			
B	b4	0.776			0.860	0.606	0.860
	b3	0.800	0.046	23.065			
	b2	0.768	0.044	22.123			
	b1	0.769	0.044	22.153			
C	c1	0.770			0.818	0.530	0.817
	c2	0.681	0.044	18.749			
	c3	0.779	0.048	21.386			
	c4	0.675	0.045	18.592			
D	d1	0.860			0.887	0.663	0.887
	d2	0.783	0.035	26.286			
	d3	0.817	0.034	27.922			
	d4	0.796	0.035	26.887			
F	f4	0.704			0.835	0.560	0.835
	f3	0.799	0.057	20.203			
	f2	0.723	0.055	18.600			
	f1	0.763	0.057	19.483			
G	g1	0.896			0.924	0.753	0.924
	g2	0.833	0.028	32.910			
	g3	0.884	0.026	37.224			
	g4	0.856	0.027	34.773			

Variable codes in the model correspond to the following constructs: A: Digital Literacy, B: Ecological Values, C: Behavioral Attitude, D: Subjective Norms, F: Perceived Behavioral Control, G: Green Food Purchase Intention.

Discriminant validity is generally tested by comparing the AVE value of confirmatory factor analysis with the results of correlation analysis. Discriminant validity is mainly tested by comparing the internal correlation coefficient of the dimension with the external correlation coefficient. When the coefficient within the dimension is greater than the external coefficient, discriminant validity is obvious.

Discriminant validity was evaluated using the Fornell-Larcker criterion. As presented in Table 5, the square root of the AVE for each construct (shown on the diagonal) was greater than its highest correlation with any other construct, thus confirming that the constructs were distinct from one another.

**Table 5:** Analysis of discriminant validity of latent facet indicators.

Latent constructs	G	F	D	C	B	A
G	0.868					
F	0.626	0.748				
D	0.517	0.405	0.814			
C	0.662	0.531	0.478	0.728		
B	0.606	0.510	0.365	0.525	0.778	
A	0.609	0.543	0.203	0.530	0.444	0.826

Variable codes in the model correspond to the following constructs: A: Digital Literacy, B: Ecological Values, C: Behavioral Attitude, D: Subjective Norms, F: Perceived Behavioral Control, G: Green Food Purchase Intention.

#### 4.1.2 Convergent validity

When using confirmatory factor analysis for convergent validity analysis, the standardized loading coefficient is used to indicate the correlation between the latent factor and the observed variable. Generally, the standardized loading coefficient should be greater than 0.5. Items with a value below 0.5 cannot fully explain the item and should be deleted. Furthermore, the significance *p*-value of the standardized loading coefficient should be less than 0.05 to demonstrate a correlation between the latent factor and the observed variable. According to table, the standardized loading coefficients for the scale items in this survey were all greater than 0.6, which is acceptable level.

Convergent validity is typically tested using metrics such as Convergent Factor Analysis (CFA) CR and AVE. The CR is often used to test the internal consistency of a facet, which is similar to the Cronbach’s alpha. A higher CR indicates greater internal consistency and convergence, with a typical critical value > 0.7. The AVE reflects the average explanatory power of the latent factor for the observed variables. A higher AVE indicates higher convergent validity, with a typical critical value > 0.5.

The data collected in this paper were run in Amos 28.0 software. Standardized loading coefficients were displayed in the Estimates option in the output window. After data organization and related calculations, the data are summarized in table. This study conducted CFA analyses on the seven facets. Following the criteria proposed by Fornell and Larcker (103), all factor loadings in this study exceeded 0.6 with significant *p*-values, while CR values were above 0.7 and AVE values surpassed 0.5. These results confirm the convergent validity of all seven facets (Table 4).

Discriminant validity was assessed by comparing the square root of AVE with the correlation coefficients between the relevant latent constructs. The results showed that the square root of AVE on the bold diagonal line was higher than the correlation coefficients between the latent factor. Therefore, the measurement model has acceptable discriminant validity, supporting the discriminant validity between the constructs (Table 5).

### 4.2 Structural equation model

**Table 6:** Structural model goodness of fit table.

Index	Test results	Reference standards
CMIN/DF	3.856	1–3 is excellent, 3–5 is good (121)
GFI	0.910	>0.9 is excellent, >0.8 is good (121)
IFI	0.944	>0.9 is excellent, >0.8 is good (122)
TLI	0.936	>0.9 is excellent, >0.8 is good (101)
CFI	0.944	>0.9 is excellent, >0.8 is good (120)
RMSEA	0.058	<0.05 is excellent, <0.08 is good (120)

**Figure 2:**
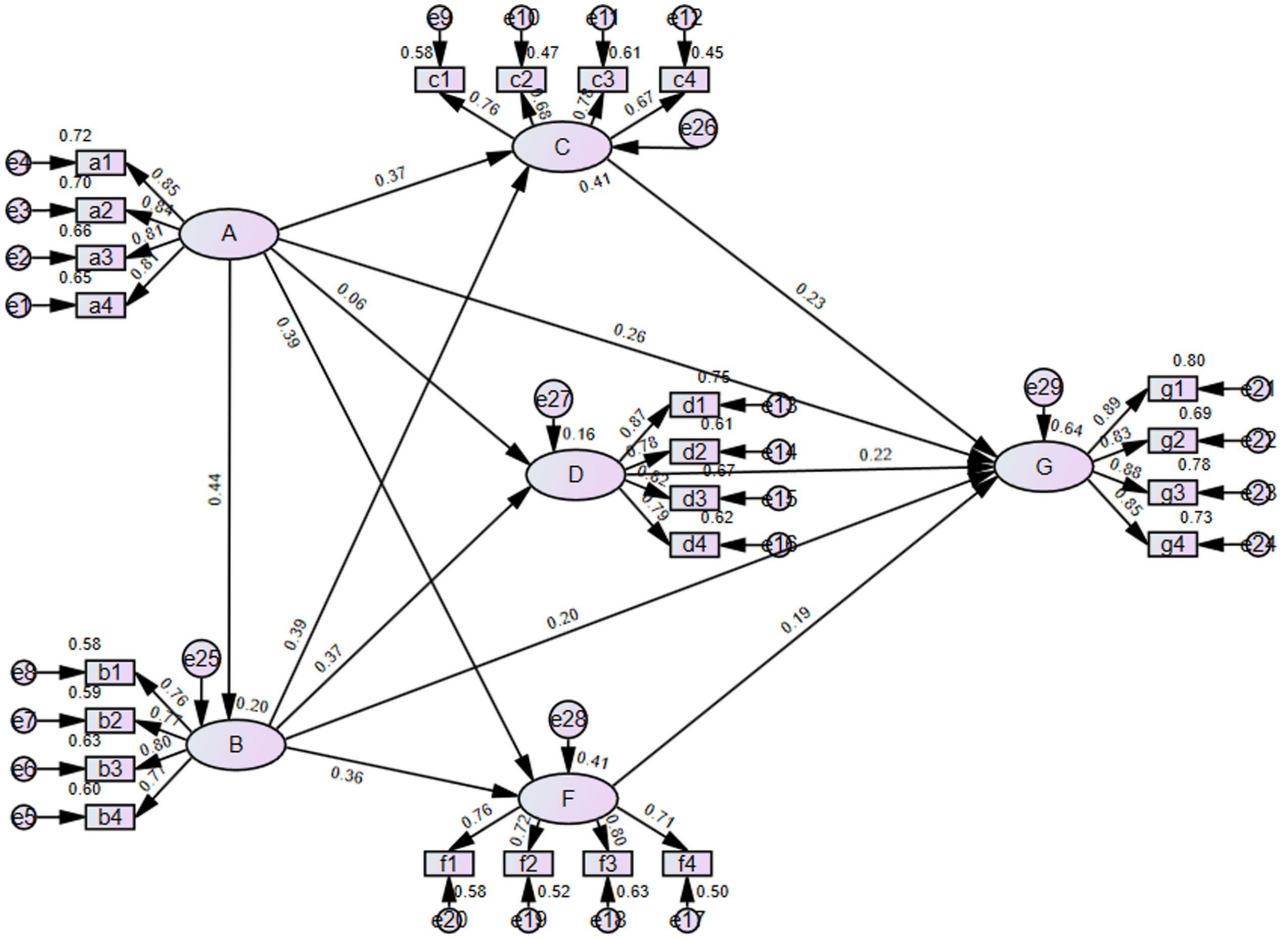
Path Diagram of the Extended TPB Model for Green Food Purchase Intention Among Chinese University Students. Variable codes in the model correspond to the following constructs: **A**: Digital Literacy, **B**: Ecological Values, **C**: Behavioral Attitude, **D**: Subjective Norms, **F**: Perceived Behavioral Control, **G**: Green Food Purchase Intention.

The evaluation of a structural model primarily relies on the significance of path coefficients or standardized factor loadings, as well as model fit indices. Using AMOS software to test the fit of the overall hypothesized model, we obtained a CMIN/DF = 1.834. Furthermore, based on the goodness-of-fit indices, we evaluated the data using GFI, RMSEA, IFI, and CFI, confirming that the model fit was good (Table 6). The specific path relationships between variables are visualized in (Figure 2). AMOS software analysis yielded the path coefficients and loading coefficients of the initial model, as shown in Table 7. The *p*-value indicates path significance.

**Table 7:** Hypothesis path test table.

Hypothesis number	Path relationship			Estimate	SE	CR	*p*
H1	G	←	C	0.257	0.043	5.918	***
H2	G	←	D	0.193	0.025	7.761	***
H3	G	←	F	0.246	0.049	4.989	***
H4	G	←	A	0.257	0.036	7.097	***
H5	C	←	A	0.317	0.036	8.905	***
H6	D	←	A	0.069	0.048	1.458	0.145
H7	F	←	A	0.292	0.031	9.395	***
H8	G	←	B	0.234	0.046	5.085	***
H9	C	←	B	0.398	0.043	9.187	***
H10	D	←	B	0.5	0.06	8.397	***
H11	F	←	B	0.317	0.037	8.557	***
H12	B	←	A	0.377	0.034	11.108	***

***p < 0.001.

Variable codes in the model correspond to the following constructs: A: Digital Literacy, B: Ecological Values, C: Behavioral Attitude, D: Subjective Norms, F: Perceived Behavioral Control, G: Green Food Purchase Intention.

#### 4.2.1 Data results

From the analysis of the above table, “G ← C “(H1), the standardized path coefficient is 0.257, which is greater than 0. The significance *p* value is *** (*p* < 0.001), less than 0.05. This indicates that C has a positive and significant impact on G, so hypothesis H1 is established.

For path “G ← D “(H2), the standardized path coefficient is 0.193, greater than 0. The significance *p* value is *** (*p* < 0.001), less than 0.05. It shows that D has a positive and significant impact on G, and hypothesis H2 is established.

For path “G ← F “(H3), the standardized path coefficient is 0.246, greater than 0. The significance *p* value is *** (*p* < 0.001), less than 0.05. This means F has a positive and significant impact on G, and hypothesis H3 is established.

For path “G ← A “(H4), the standardized path coefficient is 0.257, greater than 0. The significance *p* value is *** (*p* < 0.001), less than 0.001. It indicates that A has a positive and significant impact on G, and hypothesis H4 is established.

For path “C ← A “(H5), the standardized path coefficient is 0.317, greater than 0. The significance *p* value is *** (*p* < 0.001), less than 0.05. This shows that A has a significant positive impact on C, and hypothesis H5 is established.

For path “D ← A “(H6), the standardized path coefficient is 0.069, greater than 0. The significance *p* value is 0.145, greater than 0.05. It means A has no significant associate on D, and hypothesis H6 does not hold.

For path “F ← A “(H7), the standardized path coefficient is 0.292, greater than 0. The significance *p* value is *** (*p* < 0.001), less than 0.05. This indicates that A has a positive and significant impact on F, and hypothesis H7 is established.

For path “G ← B “(H8), the standardized path coefficient is 0.234, greater than 0. The significance *p* value is *** (*p* < 0.001), less than 0.05. It shows that B has a positive and significant impact on G, and hypothesis H8 is established.

For path “C ← B “(H9), the standardized path coefficient is 0.398, greater than 0. The significance *p* value is *** (*p* < 0.001), less than 0.05. This means B has a significant positive impact on C, and hypothesis H9 is established.

For path “D ← B “(H10), the standardized path coefficient is 0.5, greater than 0. The significance p value is *** (*p* < 0.001), less than 0.05. It indicates that B has a positive and significant impact on D, and hypothesis H10 is established.

For path “F ← B “(H11), the standardized path coefficient is 0.317, greater than 0. The significance p value is *** (*p* < 0.001), less than 0.05. This shows that B has a positive and significant impact on F, and hypothesis H11 is established.

For path “B ← A “(H12), the standardized path coefficient is 0.377, greater than 0. The significance p value is *** (*p* < 0.001), less than 0.05. It means A has a significant positive impact on B, and hypothesis H12 is established.

#### 4.2.2 Data interpretation

The analysis shows that the core TPB variables have significant direct effects on green consumption behavior (G). Specifically, behavioral attitude (C) (*β* = 0.257, *p* < 0.001) and subjective norm (D) (*β* = 0.193, *p* < 0.001) both show significant positive impacts, which confirms the establishment of hypotheses H1 and H2, respectively. Additionally, the extended variable consequence perception (F) also demonstrates a significant positive associate (*β* = 0.246, *p* < 0.001), supporting hypothesis H3. Notably, as distal variables, digital literacy (A) (*β* = 0.257, *p* < 0.001) and ecological values (B) (*β* = 0.234, *p* < 0.001) similarly exhibit significant direct associates on consumption behavior, leading to the establishment of hypotheses H4 and H8.

Ecological values (B) serve as the primary antecedent for forming positive behavioral attitude (C) (*β* = 0.398, *p* < 0.001) and perceiving strong subjective norm (D) (*β* = 0.5, *p* < 0.001), which confirms hypotheses H9 and H10. Digital literacy (A) significantly contributes to the formation of positive behavioral attitude (C) (*β* = 0.317, *p* < 0.001) and profound consequence perception (F) (*β* = 0.292, *p* < 0.001), thus establishing hypotheses H5 and H7. However, the path from digital literacy to subjective norm did not reach significance (*β* = 0.069, *p* = 0.145), resulting in the rejection of hypothesis H6, suggesting that sources of social pressure may be more focused on real-life social circles.

The model reveals a fundamental pathway: digital literacy (A) has a significant positive impact on ecological values (B) (*β* = 0.377, *p* < 0.001), which strongly supports hypothesis H12. This finding highlights that in the information age, digital literacy is not merely a tool for accessing information but also an important pathway for cultivating individuals’ intrinsic ecological values. The research findings confirm that beyond the classic TPB pathways, digital literacy and ecological values constitute the dual foundation driving green consumption among college students.

## 5 Discussion

This study systematically expands the explanatory dimension of TPB in digital consumption scenarios by constructing the DEBF. Based on multi-source data verification from 836 college students, the study reveals the differential associate mechanisms of digital literacy and ecological values on green food consumption behavior. Among these, the falsification of H6 (digital literacy → subjective norms) provides key empirical evidence for understanding norm reconstruction under technology mediation. The following sections elaborate on the findings, theoretical implications, and research limitations.

### 5.1 Validation of core TPB constructs in the digital context

The data confirms the fundamental role of TPB’s core variables. Behavioral attitude (*β* = 0.257, *p* < 0.001), subjective norms (*β* = 0.193, *p* < 0.001), and perceived behavioral control (*β* = 0.246, *p* < 0.001) all significantly influence green consumption intentions. This finding is consistent with the theoretical resilience, even within the complex digital food environments navigated by contemporary university students.

### 5.2 The dual pathways of digital literacy: cognitive capital and enabler

Moving beyond the traditional model, Digital literacy exhibits a multifaceted influence, forming a dual driving mechanism. It not only exerts a direct effect with purchase intention (*β* = 0.257, *p* < 0.001) but also operates through significant indirect pathways by strengthening behavioral attitude (*β* = 0.317, *p* < 0.001) and enhancing perceived behavioral control (*β* = 0.292, *p* < 0.001). This suggests that digital competence may have transcended its instrumental attributes and could be becoming a structural element—a form of cognitive capital. This finding may create a theoretical tension with early studies that simplified digital literacy into technology adaptation factors (104) but aligns strongly with the core proposition of digital capability cognitive transformation theory (105, 106).

### 5.3 The systematic drive of ecological values and the lens of nature quotient (NQ)

Ecological values exhibit a more pervasive and systematic driving logic. Here is the revised text that separates empirical findings from theoretical interpretation: Their direct impact on consumption intention (H8), coupled with the triple mediation path through attitude (H9), norms (H10), and control (H11), forms a “value-behavior” transformation chain. This finding aligns with the core proposition of the VBN. Furthermore, the results suggest that digital technology may enable the externalization of value-driven behavior (Path B ← A) by facilitating a more efficient formation of pro-environmental values through informational entropy reduction (107).

To further deepen the interpretation of this value-behavior link, we introduce the concept of NQ. NQ refers to an individual’s innate affinity and connection to the natural world, representing a capacity to understand, appreciate, and connect with the natural environment (108). Our finding that ecological values powerfully shape behavioral attitudes (H9) can be enriched by this concept. Students with a higher NQ are likely to possess a more profound intrinsic connection to nature, which acts as a psychological amplifier for the translation of abstract ecological values into concrete positive attitudes toward green food (109). This aligns with the perspective of achieving “peace with nature” as a foundational element for sustainable societies, where a strong personal connection to nature underpins pro-environmental actions (110). The integration of NQ thus suggests that fostering such a connection, alongside teaching factual knowledge, could be a potent target for educational interventions aimed at promoting sustainable diets.

A counterintuitive finding of this study is the non-significant direct impact of digital literacy on subjective norms (H6). This paradox might be insightfully explained through the BMF (111). The BMF conceptualizes the mind as a sponge that actively absorbs information fitting its core values (the “comfort zone”) and expels incompatible information, a process governed by Bayesian probability-based reasoning (112, 113).

In the context of our study, digitally literate students are not passive recipients of social norms. Instead, they engage in a dynamic process of information filtering and reassessment, consistent with the mindsponge mechanism observed in the integration of socio-cultural values (114). Through this process, they may create algorithmic echo chambers that prioritize sustainability content, while simultaneously developing critical cognition toward “green performance” and greenwashing marketing on social media. This critical filtering weakens the penetrative efficiency of external, traditional social pressures from peers and family. Consequently, in algorithmically mediated environments, the traditional linear transmission of normative influence is deconstructed. The BMF thus may offer a coherent explanation for why digital literacy, rather than strengthening perceived social norms, may lead to their re-evaluation and the formation of more personalized, internally validated normative standards.

### 5.4 Theoretical contribution: the integrated digital-ecological behavior framework

The primary theoretical novelty of this study lies in its substantive reframing of the TPB within the DEBF. We move beyond a general application of TPB to demonstrate how its core constructs are reconfigured in the digital age. The DEBF context reveals that digital literacy functions as cognitive capital, actively reshaping attitude formation and redefining perceived behavioral control by equipping individuals with tools to overcome barriers.

Simultaneously, the DEBF illuminates the critical paradox of subjective norms, explained above via BMF. It underscores that we are not discussing a general TPB, but a TPB whose very mechanisms are being transformed. Ultimately, by integrating ecological values (conceptually linked to NQ) as a foundational driver, the DEBF provides a more holistic explanation for the formation of purchase intention, addressing the complex interplay of motivations that drive sustainable consumption.

### 5.5 Cultural context and health motivations

Furthermore, the strong correlation between ecological values and health attitudes (H9) in this study is not only a reflection of widespread environmental awareness but is also deeply rooted in China’s long-standing food culture. Chinese consumers, including the younger generation, have always valued the concepts of “medicine and food sharing the same origin,” “freshness,” and “food safety.” The “natural, pollution-free, and healthy” attributes represented by green food resonate deeply with these traditional cultural preferences (115, 116). Therefore, choosing green food is not only a manifestation of modern health concepts but also an affirmation and practice of traditional dietary wisdom (117).

### 5.6 Limitations and future research

This study has several limitations that should be acknowledged. First and foremost, the cross-sectional nature of the data means that the relationships observed are associative rather than causal. While the proposed model is grounded in theory and the findings are consistent with our hypotheses, the temporal precedence and causal directions implied (e.g., that digital literacy leads to changes in ecological values) cannot be definitively established. Therefore, the paths in our model should be interpreted as significant correlations that support, but do not prove, the proposed theoretical mechanisms. Second, the sample consists predominantly of university students in China, which may limit the generalizability of the findings to other populations or cultural contexts. Future research should adopt longitudinal or experimental designs to better test the causal relationships among these variables. Future research could adopt longitudinal or experimental designs. Additionally, the geographical scope and the operational definitions of constructs involve a degree of subjectivity. Other potential influencing factors, such as financial status and price sensitivity, were not incorporated, which is a key consideration given students’ limited disposable income and the typical price premium of green food.

Future research should explicitly incorporate moderating variables like price sensitivity. It could also leverage consumption big data to track behavioral dynamics, extend into emerging digital marketing scenarios (e.g., short videos and live streams), and test the DEBF’s applicability in different cultural contexts to evaluate its global potential.

## 6 Implications

Based on the findings of this study, we derive a set of implications across theoretical, practical, and policy domains, which also inform future research directions.

### 6.1 Theoretical implications

This study makes several significant contributions to the literature on sustainable consumption behavior.

Theorizing the Role of Digital Competence: Our DEBF moves beyond treating technology as a mere external tool or context. It endogenizes digital literacy within the TPB framework, theorizing it as a form of cognitive capital that actively reshapes the core psychological building blocks of behavior—specifically, by reinforcing positive attitudes and enhancing perceived behavioral control. This provides a more nuanced understanding of how individuals navigate and leverage digital environments to facilitate sustainable choices.

Articulating the Motivational Mechanism of Values: The study confirms the tripartite anchoring effect with ecological values on the TPB’s antecedent constructs. It demonstrates that ecological values are not just a distal background factor but a proximal, systematic motivator that strengthens attitudes, amplifies perceived social norms, and bolsters the sense of control over green purchasing. This offers a new paradigm for explaining the individual heterogeneity observed in green consumption.

Challenging Norm Transmission in the Digital Age: The non-significant path from digital literacy to subjective norms reveals a paradox of digitally empowered autonomy. This finding challenges the traditional linear model of social influence in TPB, suggesting that in algorithm-driven, decentralized digital networks, the mechanisms of norm transmission and internalization may be undergoing a fundamental transformation. This invites a theoretical reconceptualization of the “subjective norms” construct itself.

### 6.2 Practical and managerial implications

For businesses, marketers, and platform operators aiming to promote green food, our findings suggest the value of segmented and targeted strategies.

Engaging the Digitally Literate: For consumers with high digital literacy, enhance decision support by integrating interactive tools directly into e-commerce platforms. Examples include carbon footprint trackers, blockchain-based food traceability systems, and smart filters that can sort products by multiple sustainability certifications. This appeals to their cognitive engagement and empowers their perceived control.

Motivating the Ecologically Driven: For consumers driven by strong ecological values, foster a sense of community and normative pressure through virtual green consumption communities, user-generated content (UGC) campaigns, and influencer partnerships that highlight authentic sustainable lifestyles. Storytelling and visible impact reports can resonate deeply with their value system.

Building Trust for All: Address the “trust conflict” identified among students by ensuring transparency and credibility. This can be achieved through clear, verifiable green certifications, showcasing authentic production stories via short videos, and encouraging detailed user reviews that specifically mention product quality and environmental attributes.

### 6.3 Policy implications

Policymakers and university administrators can leverage these insights to design more effective interventions.

Integrated Education: Develop and implement “digital literacy-ecological values” collaborative cultivation programs. Environmental education should be integrated into digital skills curricula in universities, teaching students not just how to find information online, but how to critically evaluate environmental claims and make informed sustainable choices.

Leveraging Digital Platforms for Public Health: Public service campaigns should collaborate with social media and e-commerce platforms. By utilizing algorithm optimization strategies, these platforms can be encouraged to prioritize the dissemination of authoritative, science-based green information and authentically sustainable products, thereby guiding young consumers toward healthier and more sustainable diets.

Supporting Affordable Access: Recognizing the financial constraints of students, policymakers could explore schemes such as subsidies for green food in university canteens or tax incentives for companies offering affordable green options targeted at the youth market. This helps bridge the intention-behavior gap caused by price sensitivity.

### 6.4 Directions for future research

Employ Longitudinal or Experimental Designs to establish stronger causal inferences among the variables, particularly regarding the dynamic relationship between digital literacy and the evolution of ecological values.

Incorporate Behavioral Measures to move beyond intention. Tracking actual purchase behavior through methods like purchase diaries or analyzing consumption data would provide a more robust test of the DEBF model and help better understand the intention-behavior gap.

Explore Additional Moderators such as price sensitivity, disposable income, or specific social media platform use to clarify the conditions under which the relationships in the DEBF are strengthened or weakened.

Test the DEBF’s Generalizability by applying it to other cultural contexts, different demographic groups (e.g., young professionals, families), and other domains of sustainable consumption (e.g., energy conservation, sustainable fashion) to evaluate its broader utility.

## 7 Conclusion

This study makes a theoretical contribution by extending TPB through the proposed DEBF to elucidate the psychological mechanisms driving green food consumption among Chinese university students. The empirical results lead to three fundamental conclusions. First, digital literacy appears to serve as a form of cognitive capital within the DEBF, potentially influencing purchase intention not only directly but also indirectly by fostering positive attitudes and enhancing perceived behavioral control. Second, ecological values may act as a pervasive motivational anchor, exerting a direct associate with intention and systematically strengthening the core antecedents of attitude, subjective norms, and perceived control. Third, the non-significant influence of digital literacy on subjective norms suggests a paradox of digital empowerment, indicating that in algorithmically mediated environments, the transmission mechanisms of traditional social norms may be undergoing a fundamental shift.

In summary, by integrating digital literacy and ecological values into the TPB paradigm, this study provides a more nuanced and contextually relevant understanding of sustainable consumption behaviors in the digital age. The DEBF demonstrates that the classic TPB model is not replaced, but rather meaningfully reconfigured when accounting for the interplay between technological competencies and intrinsic values.

## Data Availability

The original contributions presented in the study are included in the article/Supplementary material, further inquiries can be directed to the corresponding authors.

## References

[1] Hao X Li Y Ren S Wu H Hao Y. The role of digitalization on green economic growth: does industrial structure optimization and green innovation matter? J Environ Manag. (2023) 325:116504. doi: 10.1016/j.jenvman.2022.116504, 36272290

[2] Durmaz Y Akdoğan L. The effect of environmental responsibility on green consumption intention: the moderator role of price sensitivity and the mediator role of environmental concern. A case study in Turkey. Environ Dev Sustain. (2024) 26:9089–114. doi: 10.1007/s10668-023-03083-6

[3] Jiang B Raza MY. Research on China's renewable energy policies under the dual carbon goals: a political discourse analysis. Energ Strat Rev. (2023) 48:101118. doi: 10.1016/j.esr.2023.101118

[4] Jones AD Hoey L Blesh J Miller L Green A Shapiro LF. A systematic review of the measurement of sustainable diets. Adv Nutr. (2016) 7:641–64. doi: 10.3945/an.115.011015, 27422501 PMC4942861

[5] Lucas E Guo M Guillén-Gosálbez G. Low-carbon diets can reduce global ecological and health costs. Nat Food. (2023) 4:394–406. doi: 10.1038/s43016-023-00749-2, 37188875 PMC10208974

[6] Poore J Nemecek T. Reducing food’s environmental impacts through producers and consumers. Science. (2018) 360:987–92. doi: 10.1126/science.aaq0216, 29853680

[7] Grummon AH Lee CJY Robinson TN Rimm EB Rose D. Simple dietary substitutions can reduce carbon footprints and improve dietary quality across diverse segments of the US population. Nat Food. (2023) 4:966–77. doi: 10.1038/s43016-023-00864-0, 37884673 PMC10725296

[8] Dong C Bu X Liu J Wei L Ma A Wang T. Cardiovascular disease burden attributable to dietary risk factors from 1990 to 2019: a systematic analysis of the global burden of disease study. Nutr Metab Cardiovasc Dis. (2022) 32:897–907. doi: 10.1016/j.numecd.2021.11.012, 35067445

[9] Afshin A Sur PJ Fay KA Cornaby L Ferrara G Salama JS . Health effects of dietary risks in 195 countries, 1990–2017: a systematic analysis for the global burden of disease study 2017. Lancet. (2019) 393:1958–72. doi: 10.1016/S0140-6736(19)30041-8, 30954305 PMC6899507

[10] Michel M Eldridge AL Hartmann C Klassen P Ingram J Meijer GW. Benefits and challenges of food processing in the context of food systems, value chains and sustainable development goals. Trends Food Sci Technol. (2024) 153:104703. doi: 10.1016/j.tifs.2024.104703

[11] Hong Y Al Mamun A Masukujjaman M Yang Q. Sustainable consumption practices among Chinese youth. Humanit Soc Sci Commun. (2024) 11:1058. doi: 10.1057/s41599-024-03582-5

[12] Available online at: https://www.mckinsey.com/featured-insights/china/chinas-digital-economy-a-leading-global-force (Accessed August 3, 2017).

[13] Schlegelmilch BB. Global digital marketing strategy In: Global marketing strategy. Management for professionals. Cham: Springer (2022)

[14] Tufford AR Brennan L van Trijp H D'Auria S Feskens E Finglas P . A scientific transition to support the 21st century dietary transition. Trends Food Sci Technol. (2023) 131:139–50. doi: 10.1016/j.tifs.2022.11.021

[15] Boen H Glenisson L Hallez L Smits T. Byte into sustainability: a scoping review of digital food environment attributes that shape consumers’ sustainability perceptions, attitudes, intentions, and behaviours. Int J Behav Nutr Phys Act. (2025) 22:132. doi: 10.1186/s12966-025-01832-6, 41146148 PMC12560354

[16] Bennett R Keeble M Zorbas C Sacks G Driessen C Grigsby-Duffy L . The potential influence of the digital food retail environment on health: a systematic scoping review of the literature. Obes Rev. (2024) 25:e13671. doi: 10.1111/obr.13671, 38104965

[17] Schiro JL Shan LC Tatlow-Golden M Li C Wall P. #Healthy: smart digital food safety and nutrition communication strategies—a critical commentary. npj Sci Food. (2020) 4:14. doi: 10.1038/s41538-020-00074-z, 33083546 PMC7530665

[18] Consavage Stanley K Harrigan PB Serrano EL Kraak VI. A systematic scoping review of the literacy literature to develop a digital food and nutrition literacy model for low-income adults to make healthy choices in the online food retail ecosystem to reduce obesity risk. Obes Rev. (2022) 23:e13414. doi: 10.1111/obr.13414, 35092142 PMC9286643

[19] Ares G Natero V Gugliucci V Machín L Alcaire F de León C . Health-washing of ultraprocessed products on Instagram: prevalence and strategies in an emerging market. J Nutr Educ Behav. (2023) 55:815–22. doi: 10.1016/j.jneb.2023.09.001, 37777932

[20] Gomes S Lopes JM Nogueira S. Willingness to pay more for green products: a critical challenge for Gen Z. J Clean Prod. (2023) 390:136092. doi: 10.1016/j.jclepro.2023.136092

[21] Rozenkowska K. Theory of planned behavior in consumer behavior research: a systematic literature review. Int J Consum Stud. (2023) 47:2670–700. doi: 10.1111/ijcs.12970

[22] Shi H Wang J Huang R Zhao J Zhang Y Jiang N . Application of the extended theory of planned behavior to understand Chinese students’ intention to improve their oral health behaviors: a cross-sectional study. BMC Public Health. (2021) 21:2303. doi: 10.1186/s12889-021-12329-9, 34923971 PMC8684633

[23] Shmueli L. Predicting intention to receive COVID-19 vaccine among the general population using the health belief model and the theory of planned behavior model. BMC Public Health. (2021) 21:804. doi: 10.1186/s12889-021-10816-7, 33902501 PMC8075011

[24] Nogueira M Dias F Santos V. Sustainable mobility choices: exploring the impact of consumers' values, attitudes, perceived behavioural control and subjective norms on the likelihood to choose sustainable mobility options. J Consum Behav. (2023) 22:511–28. doi: 10.1002/cb.2144

[25] Aschwanden D Strickhouser JE Sesker AA Lee JH Luchetti M Terracciano A . Preventive behaviors during the COVID-19 pandemic: associations with perceived behavioral control, attitudes, and subjective norm. Front Public Health. (2021) 9:662835. doi: 10.3389/fpubh.2021.662835, 34026716 PMC8139398

[26] Abbad MMM. Using the UTAUT model to understand students’ usage of e-learning systems in developing countries. Educ Inf Technol. (2021) 26:7205–24. doi: 10.1007/s10639-021-10573-5, 34025204 PMC8122219

[27] Yang H Xu N Lin X Zhang W. Integrating AI literacy into the TAM-TPB model to explain students’ intention to use educational AI through MASEM approach. Comput Hum Behav Rep. (2025) 20:100833. doi: 10.1016/j.chbr.2025.100833

[28] Donald IJ Cooper SR Conchie SM. An extended theory of planned behaviour model of the psychological factors affecting commuters' transport mode use. J Environ Psychol. (2014) 40:39–48. doi: 10.1016/j.jenvp.2014.03.003

[29] Ji H Dong J Pan W Yu Y. Associations between digital literacy, health literacy, and digital health behaviors among rural residents: evidence from Zhejiang, China. Int J Equity Health. (2024) 23:68. doi: 10.1186/s12939-024-02150-2, 38594723 PMC11003150

[30] Sanchez LA Roa-Diaz ZM Gamba M Grisotto G Londono AMM Mantilla-Uribe BP . What influences the sustainable food consumption behaviours of university students? A systematic review. Int J Public Health. (2021) 66:1604149. doi: 10.3389/ijph.2021.1604149 34557062 PMC8454891

[31] Kickbusch I Piselli D Agrawal A Balicer R Banner O Adelhardt M . The lancet and financial times commission on governing health futures 2030: growing up in a digital world. Lancet. (2021) 398:1727–76. doi: 10.1016/S0140-6736(21)01824-9 34706260

[32] Rodriguez-Jimenez L Romero-Martín M Gómez-Salgado J. The emergency department carbon footprint calculator: design and validation. Medicine. (2025) 104:e41652. doi: 10.1097/MD.0000000000041652, 40441234 PMC12129524

[33] Upham P Bögel P Klapper RG Kašperová E. Theorising individual agency within sociotechnical sustainability transitions frames: a social psychological review In: Research handbook of sustainability agency. Cheltenham, UK: Edward Elgar Publishing (2021). 29–45.

[34] Farmer J Steiner A Kilpatrick S McCosker A Carlisle K Kamstra P. Value cocreation and innovation involving consumers and providers interacting with technology: a digital ethnographic study of online mental health forums. J Serv Manag. (2025) 36:270–90. doi: 10.1108/JOSM-01-2023-0029

[35] Janschitz G Penker M. How digital are ‘digital natives’ actually? Developing an instrument to measure the degree of digitalisation of university students–the DDS-index. Bull Sociol Methodol. (2022) 153:127–59. doi: 10.1177/07591063211061760

[36] Chang CW Chang SH. The impact of digital disruption: influences of digital media and social networks on forming digital natives’ attitude. SAGE Open. (2023) 13:21582440231191741. doi: 10.1177/21582440231191741

[37] He J Sui D. Investigating college students' green food consumption intentions in China: integrating the theory of planned behavior and norm activation theory. Front Sustain Food Syst. (2024) 8:1404465. doi: 10.3389/fsufs.2024.1404465

[38] Grech A Howse E Boylan S. A scoping review of policies promoting and supporting sustainable food systems in the university setting. Nutr J. (2020) 19:97. doi: 10.1186/s12937-020-00617-w, 32912299 PMC7488481

[39] Martini M Rizzo M Fedi A Loera B Gattino S. Promoting sustainable food behavior among young people: evidence-based suggestions for intervention in higher education. Int J Sustain High Educ. (2025). doi: 10.1108/IJSHE-05-2024-0334

[40] State Council of the People's Republic of China. Working guidance for carbon dioxide peaking and carbon neutrality in full and faithful implementation of the new development philosophy. (2021). Available online at: https://www.gov.cn/zhengce/2021-10/24/content_5644613.htm

[41] Song Y Qin Z Qin Z. Green marketing to Gen Z consumers in China: examining the mediating factors of an eco-label–informed purchase. SAGE Open. (2020) 10:2158244020963573. doi: 10.1177/2158244020963573

[42] Daxue Consulting. Sustainable consumption in China: is the green wave coming? (2023). Available online at: https://daxueconsulting.com/sustainable-consumption-china/

[43] Xie P Zhang Y Chen R Lin Z Lu N. Social media’s impact on environmental awareness: a marginal treatment effect analysis of WeChat usage in China. BMC Public Health. (2024) 24:3102. doi: 10.1186/s12889-024-20721-4 39574041 PMC11580196

[44] Vuong QH Nguyen MH. On nature quotient. Pac Conserv Biol. (2025) 31:PC25028. doi: 10.1071/PC25028 42231539

[45] Lengieza ML Aviste R. Relationships between people and nature: nature connectedness and relational environmental values. Curr Opin Psychol. (2025) 62:101984. doi: 10.1016/j.cresp.2024.100289 39765177

[46] Nguyen MH La VP Le TT Vuong QH. Introduction to Bayesian Mindsponge framework analytics: an innovative method for social and psychological research. MethodsX. (2022) 9:101808. doi: 10.1016/j.mex.2022.101884 36034522 PMC9400117

[47] Kumar G Nayak JK. A meta-analysis of TPB model in predicting green energy behavior: the moderating role of cross-cultural factors. J Int Consum Market. (2023) 35:147–65. doi: 10.1080/08961530.2022.2070900

[48] Kour M. Understanding the drivers of green consumption: a study on consumer behavior, environmental ethics, and sustainable choices for achieving SDG 12. SN Bus Econ. (2024) 4:97. doi: 10.1007/s43546-024-00691-w

[49] Shen X Xu Q Liu Q. Predicting sustainable food consumption across borders based on the theory of planned behavior: a meta-analytic structural equation model. PLoS One. (2022) 17:e0275312. doi: 10.1371/journal.pone.0275312, 36383540 PMC9668161

[50] Ma J Yin Z Hipel KW Li M He J. Exploring factors influencing the application accuracy of the theory of planned behavior in explaining recycling behavior. J Environ Plan Manag. (2023) 66:445–70. doi: 10.1080/09640568.2021.2001318

[51] Schneider S Beege M Nebel S Schnaubert L Rey GD. The cognitive-affective-social theory of learning in digital environments (CASTLE). Educ Psychol Rev. (2022) 34:1–38. doi: 10.1007/s10648-021-09626-5, 34226808 PMC8242289

[52] Jun K Yoon B. Consumer perspectives on restaurant sustainability: an SOR model approach to affective and cognitive states. J Foodserv Bus Res. (2024):1–24. doi: 10.1080/15378020.2024.2396687

[53] Zheng C Ling S Cho D. How social identity affects green food purchase intention: the serial mediation effect of green perceived value and psychological distance. Behav Sci. (2023) 13:664. doi: 10.3390/bs13080664 37622804 PMC10451480

[54] Du J Pan W. Examining energy saving behaviors in student dormitories using an expanded theory of planned behavior. Habitat Int. (2021) 107:102308. doi: 10.1016/j.habitatint.2020.102308

[55] Theocharis D Tsekouropoulos G. Sustainable consumption and branding for Gen Z: how brand dimensions influence consumer behavior and adoption of newly launched technological products. Sustainability. (2025) 17:4124. doi: 10.3390/su17094124

[56] Schulze M Janssen M Aschemann-Witzel J. How to move the transition to sustainable food consumption towards a societal tipping point. Technol Forecast Soc Change. (2024) 203:123329. doi: 10.1016/j.techfore.2024.123329

[57] Teng CC Chih C. Sustainable food literacy: a measure to promote sustainable diet practices. Sustain Prod Consum. (2022) 30:776–86. doi: 10.1016/j.spc.2022.01.008

[58] Sui D He J Liu K Lv X. Investigating the impact of the theory of planned behavior and food literacy on green food purchasing intentions among Chinese baby boomers, generation X, and generation Y. Sustainability. (2024) 16:10467. doi: 10.3390/su162310467

[59] Al-Swidi A Saleh RM. How green our future would be? An investigation of the determinants of green purchasing behavior of young citizens in a developing country. Environ Dev Sustain. (2021) 23:13436–68. doi: 10.1007/s10668-020-01220-z

[60] Ansu-Mensah P. Green product awareness effect on green purchase intentions of university students': an emerging market's perspective. Futur Bus J. (2021) 7:48. doi: 10.1186/s43093-021-00094-5

[61] Wang L Wang ZX Zhang Q Jebbouri A Wong PPW. Consumers' intention to visit green hotels–a goal-framing theory perspective. J Sustain Tour. (2022) 30:1837–57. doi: 10.1080/09669582.2021.1977937

[62] Saut M Saing T. Factors affecting consumer purchase intention towards environmentally friendly products: a case of generation Z studying at universities in Phnom Penh. SN Bus Econ. (2021) 1:83. doi: 10.1007/s43546-021-00085-2

[63] Xianyu Y Long H Wang Z Meng L Duan F. The impact of tea farmers' cognition on green production behavior in Jingmai Mountain: chain mediation by social and personal norms and the moderating role of government regulation. Sustainability. (2024) 16:8885. doi: 10.3390/su16208885

[64] Gelfand MJ Gavrilets S Nunn N. Norm dynamics: interdisciplinary perspectives on social norm emergence, persistence, and change. Annu Rev Psychol. (2024) 75:341–78. doi: 10.1146/annurev-psych-033020-013319, 37906949

[65] Ma L Shahbaz P Haq SU Boz I. Exploring the moderating role of environmental education in promoting a clean environment. Sustainability. (2023) 15:8127. doi: 10.3390/su15108127

[66] Mónus F. Environmental education policy of schools and socioeconomic background affect environmental attitudes and pro-environmental behavior of secondary school students. Environ Educ Res. (2022) 28:169–96. doi: 10.1080/13504622.2021.2023106

[67] Van Tonder E Fullerton S De Beer LT Saunders SG. Social and personal factors influencing green customer citizenship behaviours: the role of subjective norm, internal values and attitudes. J Retail Consum Serv. (2023) 71:103190. doi: 10.1016/j.jretconser.2022.103190

[68] Çoker EN Jebb SA Stewart C Clark M Pechey R. Perceptions of social norms around healthy and environmentally-friendly food choices: linking the role of referent groups to behavior. Front Psychol. (2022) 13:974830. doi: 10.3389/fpsyg.2022.974830, 36312106 PMC9611198

[69] Sharps MA Fallon V Ryan S Coulthard H. The role of perceived descriptive and injunctive norms on the self-reported frequency of meat and plant-based meal intake in UK-based adults. Appetite. (2021) 167:105615. doi: 10.1016/j.appet.2021.105615, 34332002

[70] Chan RC Lam MS. The relationship between perceived school climate, academic engagement, and emotional competence among Chinese students: the moderating role of collectivism. Learn Individ Differ. (2023) 106:102337. doi: 10.1016/j.lindif.2023.102337

[71] Zahid H Ali S Danish M Sulaiman MABA. Factors affecting consumers intentions to purchase dairy products in Pakistan: a cognitive affective-attitude approach. J Int Food Agribus Mark. (2024) 36:347–72. doi: 10.1080/08974438.2022.2125919

[72] Lim HR An S. Intention to purchase wellbeing food among Korean consumers: an application of the theory of planned behavior. Food Qual Prefer. (2021) 88:104101. doi: 10.1016/j.foodqual.2020.104101, 33071469 PMC7553994

[73] Arya B Chaturvedi S Bhati NS. Extending the theory of planned behaviour to predict sustainable food consumption. Environ Dev Sustain. (2024) 26:31277–300. doi: 10.1007/s10668-024-04466-z

[74] Lu J Liu Y Jing Q Zhang W. Chinese consumers' perceptions, attitude, and purchase intention of organic products. Appetite. (2025) 214:108142. doi: 10.1016/j.appet.2025.108142, 40409362

[75] Friedman VJ Wright CJ Molenaar A McCaffrey T Brennan L Lim MS. The use of social media as a persuasive platform to facilitate nutrition and health behavior change in young adults: web-based conversation study. J Med Internet Res. (2022) 24:e28063. doi: 10.2196/28063, 35583920 PMC9161050

[76] Qi X Ploeger A. An integrated framework to explain consumers’ purchase intentions toward green food in the Chinese context. Food Qual Prefer. (2021) 92:104229. doi: 10.1016/j.foodqual.2021.104229

[77] Liu C Zheng Y Cao D. An analysis of factors affecting selection of organic food: perception of consumers in China regarding weak signals. Appetite. (2021) 161:105145. doi: 10.1016/j.appet.2021.105145, 33515620

[78] Hu X Meng H. Digital literacy and green consumption behavior: exploring dual psychological mechanisms. J Consum Behav. (2023) 22:272–87. doi: 10.1002/cb.2122

[79] Zolfaghari A Thomas-Francois K Somogyi S. Consumer adoption of digital grocery shopping: what is the impact of consumer's prior-to-use knowledge? Br Food J. (2023) 125:1355–73. doi: 10.1108/BFJ-02-2022-0187

[80] Severo EA De Guimarães JCF Wanderley LSO Gueiros MMB Jabbour CJC. Influence of the COVID-19 pandemic on the use of social media on awareness' socio-environmental and sustainable consumption: consolidating lessons from the pandemic. Environ Dev. (2023) 46:100865. doi: 10.1016/j.envdev.2023.100865, 37192845 PMC10165870

[81] Hodges BH Rączaszek-Leonardi J. Ecological values theory: beyond conformity, goal-seeking, and rule-following in action and interaction. Rev Gen Psychol. (2022) 26:86–103. doi: 10.1177/10892680211048174

[82] Stern PC Dietz T Abel T Guagnano GA Kalof L. A value-belief-norm theory of support for social movements: the case of environmentalism. Hum Ecol Rev. (1999) 6:81–97.

[83] Gao H Dai X Wu L Zhang J Hu W. Food safety risk behavior and social co-governance in the food supply chain. Food Control. (2023) 152:109832. doi: 10.1016/j.foodcont.2023.109832

[84] Amin S Tarun MT. Effect of consumption values on customers' green purchase intention: a mediating role of green trust. Soc Responsib J. (2021) 17:1320–36. doi: 10.1108/SRJ-05-2020-0191

[85] Green EC Murphy EM Gryboski K. The health belief model. Hoboken, NJ, USA: The Wiley encyclopedia of health psychology (2020). 211–4.

[86] Wang Q Li Y Li R. Ecological footprints, carbon emissions, and energy transitions: the impact of artificial intelligence (AI). Humanit Soc Sci Commun. (2024) 11:1043. doi: 10.1057/s41599-024-03520-5

[87] Uhlenbrook S Yu W Schmitter P Smith DM. Optimizing the water we eat—rethinking policy to enhance productive and sustainable use of water in agri-food systems across scales. Lancet Planet Health. (2022) 6:e59–65. doi: 10.1016/S2542-5196(21)00264-3, 34998461

[88] Cozzio C Volgger M Taplin R. Point-of-consumption interventions to promote virtuous food choices of tourists with self-benefit or other-benefit appeals: a randomised field experiment. J Sustain Tour. (2022) 30:1301–19. doi: 10.1080/09669582.2021.1932936

[89] Gleaves JM Kemps E Prichard I Tiggemann M. I'll have what she's having (but not what they're having): the moderating role of group membership in the choice effect of social norms on food in an online environment. Appetite. (2024) 198:107374. doi: 10.1016/j.appet.2024.107374 38679066

[90] Carfora V Cavallo C Catellani P Del Giudice T Cicia G. Why do consumers intend to purchase natural food? Integrating theory of planned behavior, value-belief-norm theory, and trust. Nutrients. (2021) 13:1904. doi: 10.3390/nu13061904, 34205879 PMC8229563

[91] Yang Q Al Mamun A Naznen F Siyu L Makhbul ZKM. Modeling the significance of health values, beliefs and norms on the intention to consume and the consumption of organic foods. Heliyon. (2023) 9:e17487. doi: 10.1016/j.heliyon.2023.e17487, 37416654 PMC10320173

[92] Piscitelli A D'Uggento AM. Do young people really engage in sustainable behaviors in their lifestyles? Soc Indic Res. (2022) 163:1467–85. doi: 10.1007/s11205-022-02955-0

[93] Chen S Li B Zhou Q Liu H. From virtual trees to real forests: the impact of gamification affordances on green consumption behaviors in ant forest. Environ Commun. (2024) 18:525–49. doi: 10.1080/17524032.2023.2213850

[94] Bogliacino F Charris R Codagnone C Folkvord F Gaskell G Gómez C . Less is more: information overload in the labeling of fish and aquaculture products. Food Policy. (2023) 116:102435. doi: 10.1016/j.foodpol.2023.102435

[95] Sundar A Cao E Wu R Kardes FR. Is unnatural unhealthy? Think about it: overcoming negative halo effects from food labels. Psychol Mark. (2021) 38:1280–92. doi: 10.1002/mar.21485

[96] Hwang J Tung T Cho H. Why do consumers leave fast fashion stores? Role of shoppers' confusion. J Fashion Mark Manag. (2023) 28:186–207. doi: 10.1108/JFMM-04-2022-0080

[97] Zhao L Zhang Y Zhang H. Research on the impact of digital literacy on farmer households' green cooking energy consumption: evidence from rural China. Int J Environ Res Public Health. (2022) 19:13464. doi: 10.3390/ijerph192013464, 36294039 PMC9603365

[98] Gong S Sun Z Wang B Yu Z. Could digital literacy contribute to the improvement of green production efficiency in agriculture? SAGE Open. (2024) 14:21582440241232789. doi: 10.1177/21582440241232

[99] Wang C Zhang XE Teng X. How to convert green entrepreneurial orientation into green innovation: the role of knowledge creation process and green absorptive capacity. Bus Strat Environ. (2023) 32:1260–73. doi: 10.1002/bse.3187

[100] Yang F Yao R Ren Y Guo L. Harmony in diversity: digital literacy research in a multidisciplinary landscape. Comput Educ. (2025) 230:105265. doi: 10.1016/j.compedu.2025.105265, 41290285

[101] Ford JK MacCallum RC Tait M. The application of exploratory factor analysis in applied psychology: a critical review and analysis. Pers Psychol. (1986) 39:291–314. doi: 10.1111/j.1744-6570.1986.tb00583.x

[102] Bagozzi RP Yi Y. On the evaluation of structural equation models. JAMS. (1988) 16:74–94. doi: 10.1007/BF02723327

[103] Fornell C Larcker DF. Evaluating structural equation models with unobservable variables and measurement error. J Mark Res. (1981) 18:39–50. doi: 10.1177/002224378101800104

[104] Helgeson J Glynn P Chabay I. Narratives of sustainability in digital media: an observatory for digital narratives. Futures. (2022) 142:103016. doi: 10.1016/j.futures.2022.103016

[105] Kai W. Social and cultural capital and learners’ cognitive ability: issues and prospects for educational relevance, access and equity towards digital communication in China. Curr Psychol. (2023) 42:15549–63. doi: 10.1007/s12144-021-02517-6

[106] Farias-Gaytan S Aguaded I Ramirez-Montoya MS. Digital transformation and digital literacy in the context of complexity within higher education institutions: a systematic literature review. Humanit Soc Sci Commun. (2023) 10:386. doi: 10.1057/s41599-023-01875-9

[107] Vuong QH La VP Nguyen MH. Informational entropy-based value formation: a new paradigm for a deeper understanding of value. Eval Rev. (2025). doi: 10.1177/0193841X251396210, 41214459

[108] Rahmani L Haasova S Czellar S Clergue V Martin C. Nature & me: promoting pro-environmental behaviors through relationship-with-nature interventions. J Environ Psychol. (2025) 102755:102755. doi: 10.1016/j.jenvp.2025.102755, 41290285

[109] Suko Y Kankaanpää R Grassini S. Adulthood nature exposure and urban living are associated with climate policy support, mediated by climate change beliefs: a structural equation modeling study. J Environ Psychol. (2025) 106:102652. doi: 10.1016/j.jenvp.2025.102652, 41290285

[110] Vuong QH La VP Ho MT Nguyen MH. Peace with nature as the new nature of peace. Visions Sustain. (2025) 24:1–25. doi: 10.13135/2384-8677/12531

[111] Vuong QH. Mindsponge theory. Poznań, Poland: Sciendo (2022).

[112] Jin R Wang X. “Somewhere I belong?” A study on transnational identity shifts caused by “double stigmatization” among Chinese international student returnees during COVID-19 through the lens of mindsponge mechanism. Front Psychol. (2022) 13:1018843. doi: 10.3389/fpsyg.2022.1018843, 36329731 PMC9623289

[113] Vuong QH Nguyen MH La VP. The mindsponge and BMF analytics for innovative thinking in social sciences and humanities. Berlin, Germany: De Gruyter (2022).

[114] Vuong QH. Global mindset as the integration of emerging socio-cultural values through mindsponge processes: a transition economy perspective 1 In: Global mindsets. London, UK: Routledge (2016). 109–26.

[115] Ballesteros-Vivas D Socas-Rodríguez B Mendiola JA Ibáñez E Cifuentes A. Green food analysis: current trends and perspectives. Curr Opin Green Sustain Chem. (2021) 31:100522. doi: 10.1016/j.cogsc.2021.100522

[116] Chen YC Tsui PL Lan BK Lee CS Chiang MC Tsai MY . The role of perceived value in shaping consumer intentions: a longitudinal study on green agricultural foods. Br Food J. (2025) 127:1343–60. doi: 10.1108/BFJ-10-2024-0987

[117] Suarez C Adibi S. Integrating traditional nutritional wisdom into digital nutrition platforms: toward culturally adaptive and inclusive health technologies. Nutrients. (2025) 17:1978. doi: 10.3390/nu17121978, 40573089 PMC12196328

[118] Reddy P Chaudhary K Sharma B Hussein S. Essaying the design, development and validation processes of a new digital literacy scale. Online Inf Rev. (2023) 47:371–97. doi: 10.1108/OIR-10-2021-0532

[119] Van de Velde L Verbeke W Popp M Buysse J Van Huylenbroeck G. Perceived importance of fuel characteristics and its match with consumer beliefs about biofuels in Belgium. Energy Policy. (2009) 37:3183–93. doi: 10.1016/j.enpol.2009.04.022

[120] Ha HY Janda S. Predicting consumer intentions to purchase energy-efficient products. J Consum Market. (2012) 29:461–9. doi: 10.1108/07363761211274974

[121] Yeung RM Morris J. An empirical study of the impact of consumer perceived risk on purchase likelihood: a modeling approach. Int J Consum Stud. (2006) 30:294–305. doi: 10.1111/j.1470-6431.2006.00493.x

[122] Rausch T M Kopplin C S. Bridge the gap: consumers' purchase intention and behavior regarding sustainable clothing. J Clean Prod. (2021) 278:123882. doi: 10.1016/j.jclepro.2020.123882

[123] Martynova E West SG Liu Y. Review of principles and practice of structural equation modeling. Struct Equ Model. (2018) 25:325–9. doi: 10.1080/10705511.2017.1401932

[124] Bentler PM. Comparative fit indices in structural equation modeling. Psychol Bull. (1990) 107:238–46. 2320703 10.1037/0033-2909.107.2.238

[125] Tucker LR Lewis C. A reliability coefficient for maximum likelihood factor analysis. Psychometrika. (1973) 38:1–10. doi: 10.1007/BF02291170

