# Supplementary material for: Digital literacy, ecological values, and green food consumption: an extended Theory of Planned Behavior model in Chinese universities
Source: Front Public Health. 2025 Dec 10;13:1723436. doi: 10.3389/fpubh.2025.1723436 (PMC12727889; doi:10.3389/fpubh.2025.1723436)

**Appendix 1 Total variance explained**

| Total variance explained | | | | | | | | | |
| --- | --- | --- | --- | --- | --- | --- | --- | --- | --- |
| become  point | Initial eigenvalues | | | Extracting the sum of squares of loadings | | | Sum of squares of rotating loads | | |
|  | total | Percentage of variance | accumulation% | total | Percentage of variance | accumulation% | total | Percentage of variance | accumulation% |
| 1 | 9.476 | 39.485 | 39.485 | 9.476 | 39.485 | 39.485 | 3.166 | 13.19 | 13.19 |
| 2 | 2.354 | 9.807 | 49.293 | 2.354 | 9.807 | 49.293 | 3.129 | 13.036 | 26.227 |
| 3 | 1.617 | 6.737 | 56.03 | 1.617 | 6.737 | 56.03 | 2.876 | 11.981 | 38.208 |
| 4 | 1.426 | 5.94 | 61.97 | 1.426 | 5.94 | 61.97 | 2.743 | 11.428 | 49.635 |
| 5 | 1.14 | 4.749 | 66.719 | 1.14 | 4.749 | 66.719 | 2.684 | 11.184 | 60.819 |
| 6 | 1.09 | 4.542 | 71.261 | 1.09 | 4.542 | 71.261 | 2.506 | 10.442 | 71.261 |
| 7 | 0.753 | 3.136 | 74.396 |  |  |  |  |  |  |
| 8 | 0.617 | 2.571 | 76.968 |  |  |  |  |  |  |
| 9 | 0.547 | 2.278 | 79.246 |  |  |  |  |  |  |
| 10 | 0.507 | 2.114 | 81.359 |  |  |  |  |  |  |
| 11 | 0.489 | 2.039 | 83.399 |  |  |  |  |  |  |
| 12 | 0.456 | 1.899 | 85.297 |  |  |  |  |  |  |
| 13 | 0.421 | 1.756 | 87.053 |  |  |  |  |  |  |
| 14 | 0.385 | 1.603 | 88.656 |  |  |  |  |  |  |
| 15 | 0.369 | 1.538 | 90.194 |  |  |  |  |  |  |
| 16 | 0.34 | 1.415 | 91.609 |  |  |  |  |  |  |
| 17 | 0.317 | 1.321 | 92.93 |  |  |  |  |  |  |
| 18 | 0.313 | 1.303 | 94.233 |  |  |  |  |  |  |
| 19 | 0.296 | 1.232 | 95.465 |  |  |  |  |  |  |
| 20 | 0.261 | 1.086 | 96.55 |  |  |  |  |  |  |
| 21 | 0.24 | 0.998 | 97.548 |  |  |  |  |  |  |
| 22 | 0.235 | 0.98 | 98.528 |  |  |  |  |  |  |
| 23 | 0.194 | 0.81 | 99.338 |  |  |  |  |  |  |
| 24 | 0.159 | 0.662 | 100 |  |  |  |  |  |  |

**Appendix 2 Rotated component matrix a**

| The rotated component matrix a | | | | | | |
| --- | --- | --- | --- | --- | --- | --- |
|  | Ingredients 1 | Ingredient 2 | Ingredient 3 | Ingredient 4 | Ingredient 5 | Ingredient 6 |
| a1 | 0.823 |  |  |  |  |  |
| a2 | 0.817 |  |  |  |  |  |
| a3 | 0.783 |  |  |  |  |  |
| a4 | 0.764 |  |  |  |  |  |
| d1 |  | 0.836 |  |  |  |  |
| d3 |  | 0.81 |  |  |  |  |
| d4 |  | 0.788 |  |  |  |  |
| d2 |  | 0.772 |  |  |  |  |
| b1 |  |  | 0.798 |  |  |  |
| b3 |  |  | 0.78 |  |  |  |
| b4 |  |  | 0.758 |  |  |  |
| b2 |  |  | 0.729 |  |  |  |
| g4 |  |  |  | 0.768 |  |  |
| g2 |  |  |  | 0.756 |  |  |
| g3 |  |  |  | 0.729 |  |  |
| g1 |  |  |  | 0.69 |  |  |
| f3 |  |  |  |  | 0.751 |  |
| f4 |  |  |  |  | 0.735 |  |
| f1 |  |  |  |  | 0.732 |  |
| f2 |  |  |  |  | 0.709 |  |
| c4 |  |  |  |  |  | 0.749 |
| c3 |  |  |  |  |  | 0.74 |
| c2 |  |  |  |  |  | 0.696 |
| c1 |  |  |  |  |  | 0.633 |

Appendix 3. Confirmatory Factor Analysis


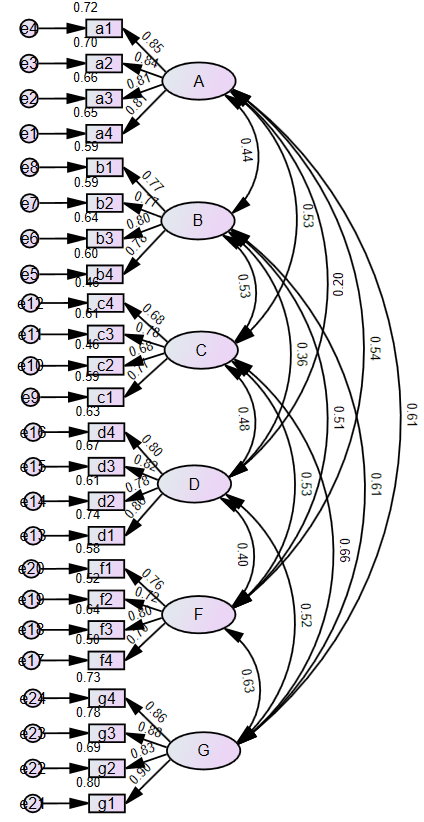

Supplement: Supplementary file 1 [file Data_Sheet_1.docx]
